# Influence of Different Pigment Incorporation Methods on the Sorption and Solubility of Medical Silicones

**DOI:** 10.1055/s-0040-1716598

**Published:** 2020-10-01

**Authors:** Adhara Smith Nobrega, Clóvis Lamartine de Moraes Melo Neto, André Pinheiro de Magalhães Bertoz, André Luiz de Melo Moreno, Marcelo Coelho Goiato

**Affiliations:** 1 Department of Dental Materials and Prosthodontics, São Paulo State University, School of Dentistry, Araçatuba, São Paulo, Brazil; 2 Department of Pediatric and Social Dentistry, São Paulo State University, School of Dentistry, Araçatuba, São Paulo, Brazil

**Keywords:** maxillofacial prosthesis, solubility, silicone elastomers, A-2186

## Abstract

**Objective** The aim of this study is to verify the influence of three pigment incorporation methods (conventional, mechanical, and industrial) on the sorption and solubility of the MDX4-4210 and A-2186 silicones.

**Materials and Methods** The groups formed were based on the silicones used (A-2186 and MDX4-4210), intrinsic pigments (pink, bronze, and black), and pigment incorporation methods (conventional, mechanical, and industrial). The dimensions of all samples were 45-mm diameter (ø) × 1-mm thickness. Readings were taken initially and after 1,008 hours of aging.

**Statistical Analysis** Three-way analysis of variance and the Tukey's test were performed (α = 0.05).

**Results** For sorption and solubility, there was no difference between the incorporation methods for the A-2186 silicone, regardless of the pigment used ( *p* > 0.05). For pink MDX4-4210, the industrial and mechanical methods showed higher values of sorption compared with the conventional method ( *p* < 0.05). For bronze MDX4-4210, the industrial method showed a higher sorption value compared with the conventional and mechanical methods ( *p* < 0.05). For black MDX4-4210, there was no difference between incorporation methods based on sorption ( *p* > 0.05). For pink MDX4-4210, the mechanical method showed a higher solubility value compared with the industrial and conventional methods ( *p* < 0.05). For black MDX4-4210 and bronze MDX4-4210, there was no statistically significant difference between incorporation methods based on solubility (p > 0.05).

**Conclusion** Based on sorption and solubility, for the A-2186 silicone, the conventional, mechanical, and industrial methods of pigment incorporation were equivalent. For the MDX4-4210 silicone, its results of sorption and solubility were varied, and further studies are recommended.

## Introduction

Facial defects can be the result of a congenital anomaly, surgical resection of a tumor, and trauma or a combination of these factors. 1 Maxillofacial prostheses may be a treatment option for patients with orofacial defects. 2 These prostheses have the function of protecting the tissues under it; in addition to returning facial aesthetics, high esteem, and quality of life for the patient. 3 Silicone is a material widely used in the manufacture of maxillofacial prostheses. 1 2 3 4 5 6 7 8

The degradation of the physical and mechanical properties of a silicone prosthesis is related to exposure to ultraviolet rays, air pollution, humidity, improper cleaning, and daily handling. 1 3 4 The literature reports that the replacement of a silicone prosthesis occurs from 3 to 12 months after its manufacture due to its degradation. 3 4 6 According to Mitra et al, one of the main reasons for replacing a maxillofacial prosthesis is due to the color change. 6 In addition, Goiato et al reported that tear strength is one of the most important properties for the durability of a silicone prosthesis. 5 Therefore, tearing of a silicone prosthesis can also be a reason for replacing this type of prosthesis. The MDX4-4210 and A-2186 silicones are examples of products widely used for the manufacture of maxillofacial prostheses. 3 4 6 7 9

The water sorption and solubility tests evaluate the process of water gain and loss of soluble components to the environment. 10 The sorption of a material represents the amount of water adsorbed on the surface and absorbed by the material body. 10 Therefore, in the sorption process, both adsorption and absorption occur simultaneously. 10 Solubility is represented by the solubilization of soluble compounds in a material. 10 Therefore, any weight loss of a material is a measure of its solubility. 10

A silicone prosthesis may be in frequent contact with saliva, sweat, and/or water (due to hygiene of the prosthesis or rain). 2 Silicone is a material that can absorb water, saliva, and sweat 2 11 and shows solubility. 11 It is important to mention that these factors (absorption and solubility) can affect the physical, mechanical, and chemical properties of a polymer (e.g., silicone elastomer). 2 11 12 13 14 15 16 In addition, Hulterström et al reported that if a prosthesis (e.g., silicone prosthesis) absorbs liquids to the point that it loses its original dimensions or shows solubility, the prosthesis may lose its functionality and appearance. 11

The pigment incorporation method can influence the amount of bubbles that will be incorporated into a silicone. 11 In the literature, three pigment incorporation methods can be observed, and these methods are classified as industrial, mechanical, and conventional (manual). 4 5 8 In the literature, few studies have compared pigment incorporation methods; 4 5 8 in addition, there are no studies comparing the influence of these methods on the sorption and solubility of medical silicones. Therefore, the objective of this study was to verify the influence of three pigment incorporation methods (industrial, mechanical, and conventional) on the sorption and solubility of the MDX4-4210 and A-2186 silicones. This study is the continuation of a previous study published in the European Journal of Dentistry. 4

## Materials and Methods

**Fig. 1:**
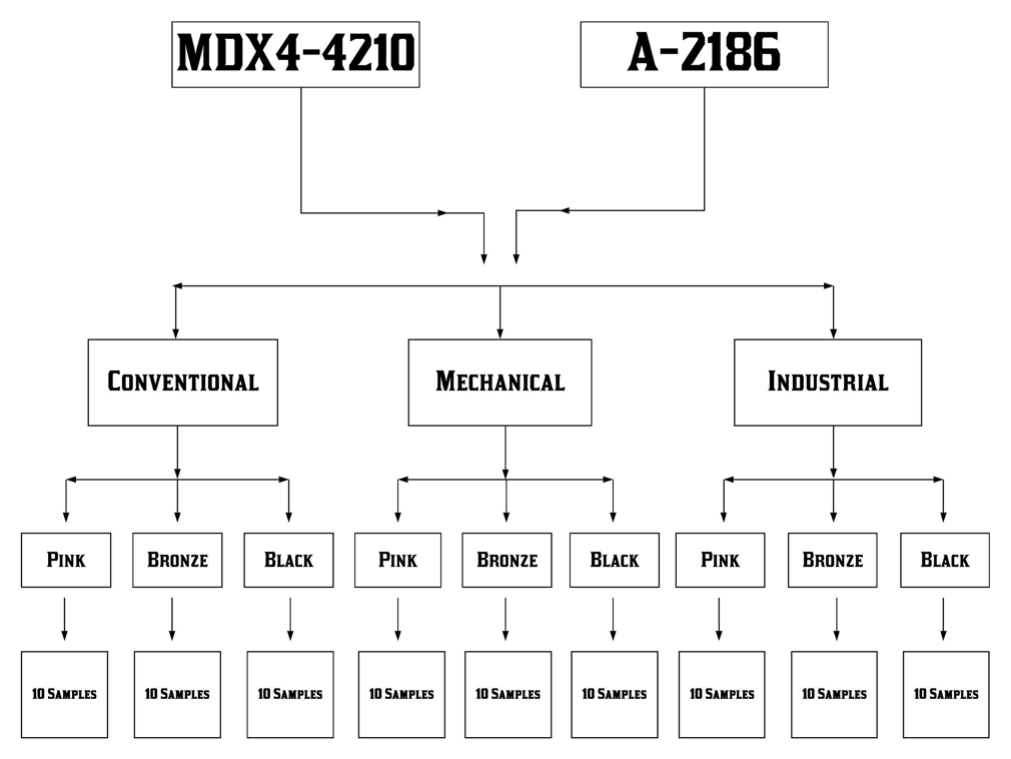
Distribution of MDX4-4210 and A-2186 groups.

The A-2186 (Factor II, United States) and MDX4-4210 (Dow Corning Corporation Medical Products, United States) silicones were used in this study to manufacture the samples. Fig. 1 shows the groups formed based on the silicones used (A-2186 and MDX4-4210), intrinsic pigments (black, bronze, and pink), and pigment incorporation methods (conventional, mechanical, and industrial). A total of 180 samples (90 samples for MDX4-4210 and 90 samples for A-2186)( *n* = 10) with dimensions of 45-mm diameter (ø) × 1-mm thickness 7 were manufactured for the sorption and solubility tests ( Fig. 1 ). All samples were made by the same operator.

Bronze (Tan FI - 215, Factor II, United States), black (Black FI - 205, Factor II, United States) and medium pink (Orbital Colors, Brazil) pigments were used. The silicones and pigments were weighed on a digital analytical balance (Adventurer, Ohaus Corporation, United States). 4 5 The bronze pigment corresponded to 0.2% of the weight of each silicone. 4 5 The black pigment also corresponded to 0.2% of the weight of each silicone. 4 5 For the pink pigment, the pigments that constituted it corresponded to 0.122% (yellow), 0.006% (black), 0.03% (red), and 0.6% TiO _2_ (opacifier) of the weight of each silicone. 4 5 With the exception of the opacifier which had a mineral origin, all other pigments used in this study had an organic origin. 4 5

For the conventional method of incorporating the pigment to the silicone, the pigment was mixed with silicone manually. 3 4 After the manipulation, the silicone was inserted into the matrix and the thickness was regularized with the aid of a metal spatula. 3 4 The matrix was closed and submitted to 1,000 Kgf for 10 minutes (Hydraulic press—Maxx, Essence Dental VH, Brazil). 5 Samples remained confined within the matrix under controlled temperature with the surface exposed for 72 hours (27 ± 2°C) to complete polymerization of the material with release of the byproduct (formaldehyde). 3 4 5 After this period, the samples were carefully separated from the matrix. 3

For the mechanical method of incorporating the intrinsic pigment to the silicone, the silicone was manually mixed to the pigment for 15 seconds, followed by a mechanical vacuum spatulation (15 psi) for 10 minutes at 425 RPM (Vacuum spatulator, Polidental, Brazil). 4 5 After this, the same steps of the conventional method were followed.

The industrial method of incorporating the pigment to the silicone was performed using a grinding machine (CHSG/3-Roll Mill, Chemieland, China). 4 5 The *Deutsches Institut für Normung* (DIN—53235) was used in this method. 4 5 After this, the same steps of the conventional method were followed. 4 5

The samples used in the sorption and solubility tests were submitted to a desiccation test according to the American Dental Association (ADA) - Specification 12 17 18 before and after accelerated aging. For this procedure, the samples remained inside a dissector (Odontobrás, Brazil) containing silica gel and at a temperature of 37 ± 2°C for 23 hours. 18 Subsequently, the samples were removed to a similar desiccator at room temperature (23°C) for 1 hour and then weighed on a precision digital scale (BEL Equipamentos Analítico, Brazil). 18 This cycle was repeated until the weight loss of each sample did not exceed more than 0.5 mg. 18 Therefore, the conditioned mass (W1) was reached when the difference between two successive readings did not exceed 0.5 mg. Posteriorly, the samples were submitted to the accelerated aging procedure, and they were weighed one more time (W2). Finally, the samples were submitted to a new dissection and final weighing (W3). The sorption and solubility were calculated according to the formulas: sorption (%) = (W2–W3)/W1 × 100; solubility (%) = (W1–W3) × 100. 17 The sorption and solubility tests were performed by the same operator.

The accelerated aging of the samples was performed in an aging chamber (Equilam, Brazil). This process was performed according to the American Society for Testing and Materials - Designation G53–96. 19 The lamps (UVB 313, 40 Watts, Equilam, Brazil) emitted ultraviolet B (UVB) light at a wavelength of 313 nm. 5 Then, they were subjected to alternating periods of UVB light and distilled water condensation saturated with oxygen under conditions of heat and 100% humidity. 3 4 5 Each aging cycle lasted 12 hours. 3 4 5 In the first 8 hours, the temperature was maintained at 60 ± 3°C, and the UVB light was imputed onto the samples. In the last 4 hours, the temperature was maintained at 45 ± 3°C and a condensation period occurred without light. 3 4 5 This test was performed for a total of 1,008 hours. 3 4 5

All data were analyzed using the Statistical Package for Social Sciences 20.0 (IBM Corp., United States). The Shapiro-Wilk statistical test was used to analyze the distribution of the numerical data. Three-way analysis of variance (ANOVA) and the Tukey's test were performed (α = 0.05).

## Results

Tables 1^2^,3,4 show the Tukey's test for sorption and solubility.

**Table 1:** Mean values (%) and standard deviation of sorption of silicones according to the incorporation method, pigment, and silicone brand

Silicone	Pigment	Incorporation method		
		Conventional	Mechanical	Industrial
A-2186	Bronze	0.07 (0.04) ^Aa^	0.04 (0.02) ^Aa^	0.05 (0.02) ^Aa^
	Black	0.06 (0.04) ^Aa^	0.06 (0.02) ^Aa^	0.04 (0.01) ^Aa^
	Pink	0.05 (0.01) ^Aa^	0.05 (0.03) ^Aa^	0.07 (0.03) ^Aa^
MDX4-4210	Bronze	0.06 (0.03) ^Aa^	0.08 (0.03) ^Aa^	0.12 (0.05) ^Ab^
	Black	0.07 (0.02) ^Aa^	0.06 (0.02) ^Aa^	0.04 (0.03) ^Ba^
	Pink	0.05 (0.04) ^Aa^	0.12 (0.11) ^Bb^	0.12 (0.07) ^Ab^

Note: Tukey's test with a level of significance of 5%. Different uppercase letters in the column denote a statistically significant difference ( *p* < 0.05). Different lowercase letters in the row denote a statistically significant difference ( *p* < 0.05).

**Table 2:** Mean values (%) and standard deviation of sorption of the different silicones according to the incorporation method, regardless of the pigment color

Incorporation method	Silicone	
	A-2186	MDX4-4210
Conventional	0.06 (0.03) ^Aa^	0.06 (0.03) ^Aa^
Mechanical	0.05 (0.03) ^Aa^	0.09 (0.07) ^Bb^
Industrial	0.05 (0.02) ^Aa^	0.09 (0.06) ^Bb^

Note: Tukey's test with a level of significance of 5%. Different uppercase letters in the column denote a statistically significant difference ( *p* < 0.05). Different lowercase letters in the row denote a statistically significant difference ( *p* < 0.05).

**Table 3:** Mean values (%) and standard deviation of solubility of silicones according to the incorporation method, pigment, and silicone brand

Silicone	Pigment	Incorporation method		
		Conventional	Mechanical	Industrial
A-2186	Bronze	1.60 (0.87) ^Aa^	1.52 (0.33) ^Aa^	2.23 (0.85) ^Aa^
	Black	1.35 (0.57) ^Aa^	1.75 (1.26) ^Aa^	2.76 (4.01) ^Aa^
	Pink	1.85 (1.00) ^Aa^	1.31 (0.66) ^Aa^	1.78 (0.66) ^Aa^
MDX4-4210	Bronze	1.87 (0.59) ^Aa^	1.66 (0.63) ^ABa^	0.53 (0.75) ^Aa^
	Black	0.11 (0.22) ^Ba^	0.31 (0.21) ^Aa^	0.74 (0.41) ^Aa^
	Pink	0.35 (0.16) ^Ba^	2.49 (5.28) ^Bb^	0.60 (0.21) ^Aa^

Note: Tukey's test with a level of significance of 5%. Different uppercase letters in the column denote a statistically significant difference ( *p* < 0.05). Different lowercase letters in the row denote a statistically significant difference ( *p* < 0.05).

**Table 4:** Mean values (%) and standard deviation of solubility of the different silicones according to the incorporation method, regardless of the pigment color

Incorporation method	Silicone	
	A-2186	MDX4-4210
Conventional	1.60 (0.83) ^Aa^	0.78 (0.87) ^ABa^
Mechanical	1.53 (0.84) ^Aa^	1.49 (3.10) ^Aa^
Industrial	2.26 (2.35) ^Aa^	0.62 (0.50) ^Bb^

Note: Tukey's test with a level of significance of 5%. Different uppercase letters in the column denote a statistically significant difference ( *p* < 0.05). Different lowercase letters in the row denote a statistically significant difference ( *p* < 0.05).

Table 1 shows the mean values (%) and the standard deviation of sorption according to the incorporation method, pigment, and silicone brand. The pigment incorporation methods were compared based on the same pigment and silicone. For the A-2186 silicone, there was no statistically significant difference between the pigment incorporation methods based on sorption, regardless of the pigment used ( *p* > 0.05; Table 1 ). For the A-2186 silicone, based on sorption and the same pigment incorporation method, there was no statistically significant difference between the pigments used ( *p* > 0.05; Table 1 ). For the pink MDX4-4210 silicone, the industrial and mechanical methods showed higher values of sorption compared with the conventional method ( *p* < 0.05; Table 1 ). For bronze MDX4-4210, the industrial method showed a higher sorption value compared with the conventional and mechanical methods ( *p* < 0.05; Table 1 ). For black MDX4-4210 silicone, there was no difference between incorporation methods based on sorption ( *p* > 0.05; Table 1 ). For the MDX4-4210 silicone, based on sorption and the same pigment incorporation method: the pink MDX4-4210 manufactured by the mechanical method showed a higher sorption value than the bronze MDX4-4210 and black MDX4-4210 manufactured by the same method ( *p* < 0.05); the black MDX4-4210 manufactured by the industrial method showed a lower sorption value than the bronze MDX4–4210, and pink MDX4–4210 manufactured by the same method ( *p* < 0.05; Table 1 ).

Table 2 shows the mean values (%) and the standard deviation of sorption according to the silicone and pigment incorporation method, and regardless of the pigment. For the A-2186 silicone, there was no difference between the incorporation methods based on sorption ( *p* > 0.05) ( Table 2 ). For the MDX4-4210 silicone, the sorption values were higher in the mechanical and industrial methods when compared with the conventional method ( *p* < 0.05; Table 2 ). The mechanical and industrial methods of the MDX4-4210 silicone showed higher sorption values than the mechanical and industrial methods of the A-2186 silicone ( *p* < 0.05; Table 2 ).

Table 3 shows the mean values (%) and the standard deviation of solubility according to the incorporation method, pigment, and silicone brand. The pigment incorporation methods were compared based on the same pigment and silicone ( Table 3 ). For the A-2186 silicone, there was no difference between the pigment incorporation methods based on solubility, regardless of the pigment used ( *p* > 0.05; Table 3 ). For the A-2186 silicone, based on solubility and the same pigment incorporation method, there was no statistically significant difference between the pigments used ( *p* > 0.05; Table 3 ). For the pink MDX4-4210 silicone, the mechanical method showed a higher solubility value compared with the industrial and conventional methods ( *p* < 0.05; Table 3 ). For black MDX4-4210 and bronze MDX4-4210, there was no statistically significant difference between incorporation methods based on solubility ( *p* > 0.05; Table 3 ). For the MDX4-4210 silicone, based on solubility and the same pigment incorporation method: the bronze MDX4-4210 manufactured by the conventional method showed a higher solubility value than the black MDX4-4210 and pink MDX4-4210 manufactured by the same method ( *p* < 0.05); the pink MDX4-4210 manufactured by the mechanical method showed a higher solubility value than the black MDX4-4210 manufactured by the same method ( Table 3 ).

Table 4 shows the mean values (%) and the standard deviation of solubility according to the silicone and pigment incorporation method, and regardless of the pigment. For the A-2186 silicone, there was no difference between the incorporation methods based on solubility ( *p* > 0.05; Table 4 ). For the MDX4-4210 silicone, the sorption value was higher in the mechanical method compared with the industrial method ( *p* < 0.05; Table 4 ). The industrial method of the A-2186 silicone showed a higher solubility value than the industrial method of the MDX4-4210 ( *p* < 0.05; Table 4 ).

## Discussion

When a polymer is exposed to a humid environment, two processes occur simultaneously: plasticizers and other soluble components are leached out of the polymer (solubility), and water is absorbed by the polymer. 12 13 The amount of water absorbed by a polymer depends a lot on its chemical structure. 14 The water absorption is mainly caused by the polar properties of the polymer molecules. 15 According to Arima et al and Garcia-Fierro and Aleman, because water interacts with the polymer chains, it can produce some of the following effects in this order: (1) reorientation and chain displacement, that is, reversible loosening or effective plasticization of the structure; (2) solvation or reversible rupture of weak interchain bonds; and (3) irreversible disruption of the polymer matrix (microvoids). 16 20 Therefore, based on these situations, water absorption and solubility can alter the physical (e.g., color), mechanical (e.g., tear strength and hardness), and chemical properties of a polymer; 2 11 12 13 14 15 16 in addition to causing dimensional changes in this type of material. 12 16 Thus, sorption and solubility can affect the durability of a silicone prosthesis.

According to Goiato et al 2019, the period of 1,008 hours of accelerated aging represents 1 year of constant use of a silicone prosthesis by a patient. 5 In the accelerated aging process, UVB rays are emitted in the silicone samples. Most polymers contain aromatic rings and C=C bonds in their structures. 4 Aromatic rings and C=C bonds can absorb UVB rays during accelerated aging. 4 According to Nobrega et al and Nobrega et al, when a polymer molecule absorbs UVB rays, this energy promotes instability in the molecular structure. 3 4 The excess energy can be transmitted by excitation from one molecule to another, allowing the first molecule to regain its stability. 3 4 In this way, affected groups can return to their original state by releasing energy in the form of longer wavelengths such as visible light or heat. 3 4 However, when this excess energy is released, a photochemical degradation occurs, contributing to molecule deterioration. 3 4 Tetteh et al can also explain a situation of the degradation of a silicone. 21 Tetteh et al reported that the weathering can induce changes in physical, mechanical, and chemical characteristics of a polymer (e.g., silicone elastomer). 21 The degradation of a polymer due to weathering is a result of a photo-oxidative attack (a combined action of oxygen and sunlight) on the chemical structure of this material. 21 The photo-oxidative degradation causes an initial formation of free radicals, reaction of free radicals with oxygen, production of polymer oxy- and peroxy- radicals, and secondary polymer radicals, resulting in chain scission. 21 In addition, a reaction of different free radicals with each other can result in crosslinking. 21 It is also important to mention that a crosslinking can occur due to the formation of bonds between existing monomers or bonds between chains.21 Based on these situations, it is possible that the degradation of a silicone due to aging can change the sorption and solubility rates of this type of material. Therefore, accelerated aging is justified to simulate the clinical degradation of a silicone.

In this study, for the A-2186 silicone, when the incorporation methods were compared, there was no statistically significant difference based on sorption or solubility, regardless of the pigment used ( Tables 1 2 3 ). The MDX4-4210 silicone did not show the same result pattern when compared with the A-2186 silicone ( Tables 1 2 3 ). This difference in the pattern of results between the two silicones may have occurred due to a possible higher filler loading and molecular weight of the dimethylsiloxane polymer from the A-2186 silicone when compared with the MDX4-4210 silicone. 5 22 In addition, a difference between these silicones based on the polar properties of their molecules may also have influenced their results. 15

In most situations, MDX4-4210 silicone showed greater sorption than the A-2186 silicone ( *p* < 0.05; Table 2 ). On the other hand, A-2186 silicone showed a trend towards greater solubility when compared with the MDX4-4210 silicone ( Table 4 ). Perhaps as mentioned earlier, the possible different characteristics between these two silicones may have caused these situations.

Based on the results of sorption and solubility of the MDX4-4210 silicone ( Tables 1 2 3 4 ), the mechanical and industrial methods did not always show the best results (lower sorption and solubility). In this study, there were situations in which the conventional method was superior (lower sorption and solubility) when compared with the mechanical and industrial methods. It was expected that the mechanical and industrial methods would always show the best results or at least equivalent results when compared with the conventional method (due to a probable situation of less of bubbles in the material, that is, less incorporation of “humidity reservoirs”). Based on these situations, it is possible to assume that, in addition to the intrinsic characteristics of the MDX4-4210 silicone that may have influenced its sorption and solubility results, the results of this study also suggest that an intrinsic pigmentation can influence the MDX4-4210 silicone, causing unexpected (as mentioned earlier) and varied sorption and solubility results (absence of a clear pattern between the results of the MDX4-4210 groups) ( Tables 1 2 3 ). This can be suggested, for example, by the fact that in the sorption evaluation, black MDX4-4210 did not show a difference between the incorporation methods ( *p* > 0.05); however, this situation was different for pink MDX4-4210 and bronze MDX4-4210, because there was a significant difference between incorporation methods in some cases ( *p* < 0.05; Table 1 ). In the solubility assessment, only pink MDX4-4210 showed a significant difference between incorporation methods ( *p* < 0.05); thus, this corroborates the fact that an intrinsic pigmentation can generate an influence on the MDX4-4210 silicone 9 ( Table 3 ). In addition, for the MDX4-4210 silicone, there were statistically significant differences between pigmentations in several cases (in each method evaluated individually), based on the values of sorption and solubility; thus, this also corroborates the fact that the MDX4-4210 silicone can be influenced by an intrinsic pigmentation 9 ( Tables 1 2 3 ). Therefore, presumably, an intrinsic pigmentation and the intrinsic characteristics of the MDX4-4210 silicone may have a greater influence on the sorption and solubility results of this silicone than a pigment incorporation method. It is worth mentioning that the sorption and solubility results of the A-2186 silicone were not affected by pigmentations or pigment incorporation methods. ( Tables 1 2 3 4 ).

Nobrega et al evaluated color and dimensional stability using the pigment incorporation methods used in this study (conventional, mechanical and industrial), for the manufacture of the MDX4-4210 and A-2186 silicones. Although all results were clinically acceptable for dimensional and color stability after aging regardless of the incorporation method used, the mechanical and industrial methods showed the best statistical results in general (lower color and dimensional change than the conventional method). 4 In addition, Goiato et al showed an equivalence between the industrial and mechanical methods based on tear strength. 5 Despite these situations, the industrial method can have a higher cost and require more time for the manufacture of a prosthesis when compared with the mechanical method. 4 5 Another important aspect is that according to Hatamleh and Watts, mixing the base and catalyst of a silicone under vacuum (mechanical method) can reduce the amount of bubbles incorporated in this material compared with the conventional method (manual). 8 It is important to mention that air bubbles inside a silicone prosthesis can affect its elasticity, elongation, tear resistance, and aesthetics. 8 In this study for sorption and solubility, the three methods were equivalent based on A-2186 silicone in all cases. For the MDX4-4210 silicone, the results were often the opposite of what would be expected. It is worth mentioning that the results of previous studies 4 5 8 regarding pigment incorporation methods cannot be disregarded in this study. Therefore, the mechanical method would be the best option for the manufacture of silicone prostheses.

A major limitation of this study is that in the literature there are no clinical acceptability criteria (ADA or International Organization for Standardization) for sorption or solubility of silicones used in maxillofacial prostheses. Therefore, the interpretation of the results of this study was more difficult, because it was not possible to know which percentage change for sorption or solubility after the aging of a silicone prosthesis, would be clinically acceptable. Another limitation is that few studies have compared pigment incorporation methods.

## Conclusion

Based on sorption and solubility for the A-2186 silicone, the conventional, mechanical, and industrial methods of pigment incorporation were equivalent. For the MDX4-4210 silicone, its results of sorption and solubility were varied, and further studies are recommended.
